# Adductor Canal Block Duration of Analgesia Successfully Prolonged With Perineural Dexmedetomidine and Dexamethasone in Addition to IPACK Block for Total Knee Arthroplasty

**DOI:** 10.7759/cureus.10566

**Published:** 2020-09-21

**Authors:** Jared Herman, Ivan Urits, Jonathan Eskander, Alan D Kaye, Omar Viswanath

**Affiliations:** 1 Anesthesiology, Mount Sinai Medical Center, Miami Beach, USA; 2 Anesthesiology, Critical Care, and Pain Medicine, Beth Israel Deaconess Medical Center, Harvard Medical School, Boston, USA; 3 Anesthesiology and Pain Medicine, Portsmouth Anesthesia Associates, Portsmouth, USA; 4 Anesthesiology, Louisiana State University Health Shreveport, Shreveport, USA; 5 Pain Management, Valley Pain Consultants - Envision Physician Services, Phoenix, USA

**Keywords:** dexmedetomidine, dexamethasone, total knee arthroplasty, regional anesthesia, adductor canal block, ipack block, pain management

## Abstract

Total knee arthroplasty (TKA) is among the most commonly performed orthopedic procedures. Controlling the pain of this patient population is essential in improving outcomes such as opioid consumption, hospital length of stay, overall function, and rehabilitation participation following their procedure. Local anesthetic infiltration of the interspace between the popliteal artery and capsule of the posterior knee, known as the IPACK block, combined with an adductor canal block (ACB) can be used to reduce pain in the challenging area of the posterior knee after knee surgery without compromising motor function of the quadriceps muscles. One limiting factor to this combination of techniques is the duration of analgesia provided. This case series demonstrates the combination of dexmedetomidine and dexamethasone (Dex-Dex) as local anesthetic adjuvants to significantly prolong the analgesic duration of ACB (in addition to IPACK block) in three patients undergoing TKA. Preoperative ACB and IPACK blocks were performed for postoperative analgesia in three TKA patients. The anesthetic mixture was 10 cc 0.2% ropivacaine combined with 25 mcg of dexmedetomidine and 5-mg preservative-free dexamethasone for the ACB, and 0.2% ropivacaine combined with 5-mg preservative-free dexamethasone was utilized for the IPACK block. Two of the patients reported experiencing four days of analgesia and one patient reported five days of analgesia following the ACB + IPACK block. Two of the patients required no opioid analgesics postoperatively. An ACB utilizing 0.75% ropivacaine has been demonstrated to provide approximately 10.8 hours of analgesia. Our series demonstrates a significantly prolonged duration of analgesia from this injectate combination. Few studies have utilized the Dex-Dex combination. The combination, however, was previously proven to safely increase the analgesic duration of a caudal block prior to hypospadias surgeries in pediatrics. More studies are needed to understand a potential synergistic effect of Dex-Dex, which could have a substantial impact on postoperative analgesia for TKA patients.

## Introduction

Total knee arthroplasty (TKA) is among the most commonly performed orthopedic procedures, with an expected increase of up to 3.5 million procedures performed by 2030 [1]. A total of 700,000 total knee replacements were performed in the year 2012 alone. Controlling the pain of this patient population is essential in improving outcomes such as opioid consumption, hospital length of stay, and overall function following their procedure. Expediting the recovery process relies on allowing these patients to comfortably participate in early rehabilitation with as little pain as safely possible. A 2009 systematic review, which evaluated the impact of regional anesthesia on TKA outcomes, demonstrated that regional anesthesia reduced postoperative pain, reduced opioid-related adverse effects, and improved patient satisfaction when compared to intravenous patient-controlled analgesia [2].

Often, it is challenging to adequately anesthetize the posterior knee through regional technique for postoperative analgesia. Local anesthetic infiltration of the interspace between the popliteal artery and capsule of the posterior knee, known as the IPACK block, can be used to reduce pain after knee surgery. The IPACK block is a novel regional technique developed to provide analgesia to this area without compromising motor function [3]. The IPACK, combined with an adductor canal block (ACB), involves solely blocking the sensory nerves of the anterior, medial, and posterior knee, preserving motor function to encourage early ambulation and expedited rehabilitation [4,5].

ACB in combination with an IPACK block is a favorable technique to control the postoperative pain following a TKA procedure and is growing in popularity. One limiting factor to this combination of techniques is the duration of analgesia provided. Prolonging the duration of analgesia that a patient receives could potentially be even more beneficial to the patient’s recovery success and quality measures. While many local anesthetic adjuvants have been explored, this case series demonstrates the combination of dexmedetomidine and dexamethasone (Dex-Dex) as adjuvants to local anesthetic to significantly and safely prolong the analgesic duration of combined ACB and IPACK in three patients undergoing TKA.

## Case presentation

Procedural technique 

**Figure 1:**
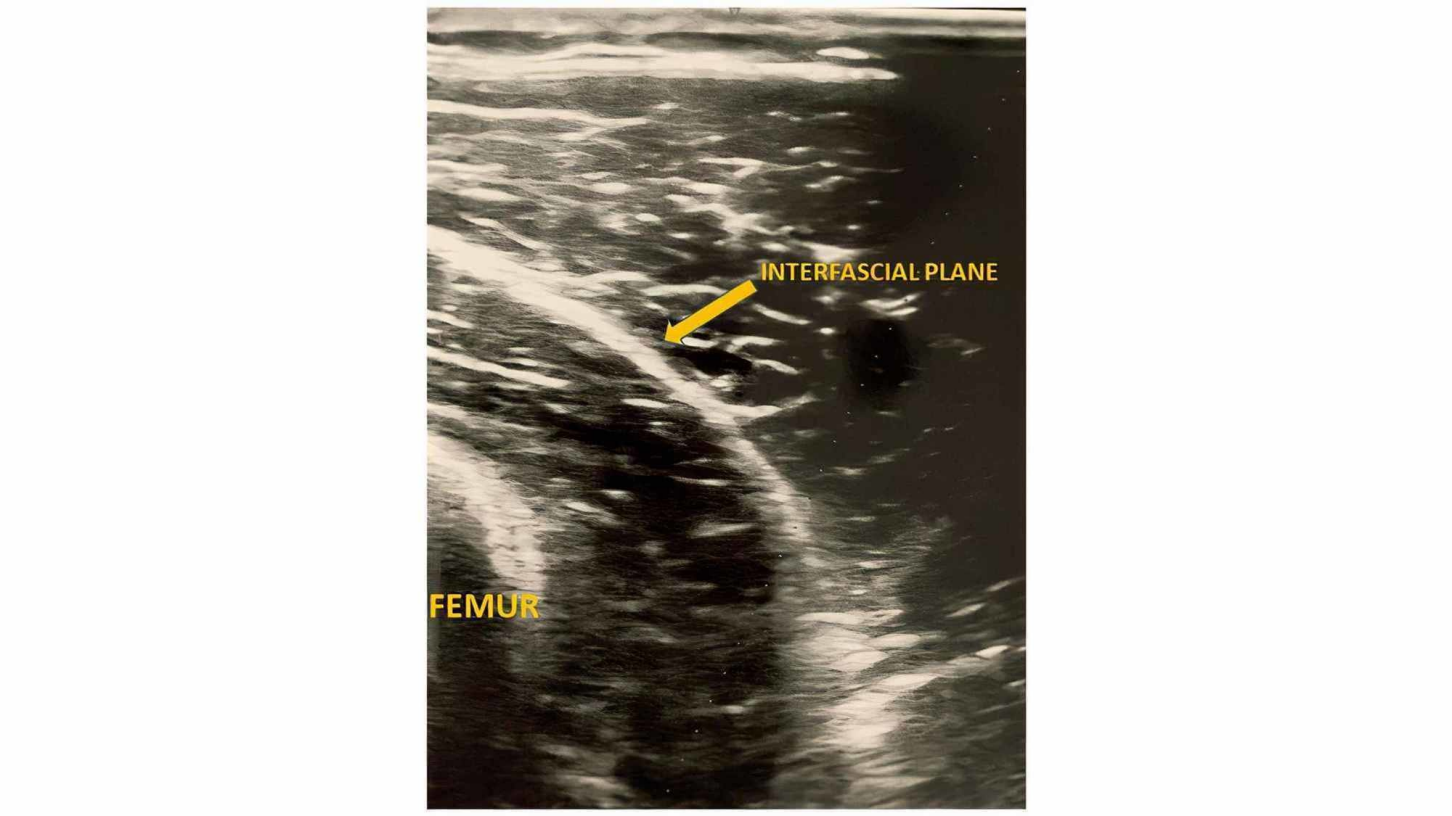
Gray-scale ultrasound with a linear probe showing the medial aspect of the posterior knee articular capsule and popliteal fossa. The interfascial plane and part of the femoral head can be seen.

Preoperatively, ACB and IPACK blocks were performed for postoperative analgesia. For the IPACK block, the patient remained in the supine position with the lower extremity flexed at the knee and abducted at the hip. To begin scanning, the transducer was placed on the lower third of the medial thigh to visualize the femur and associated blood vessels. At this point, the transducer was moved caudally to observe the femoral artery as it dives into the popliteal fossa. The transducer was then moved posteriorly and inferiorly to visualize the space between the popliteal artery and the shaft of the femur, just superior to the femoral condyles. The needle was inserted, in plane, from the anterior end of the transducer in a medial to lateral trajectory. When the needle tip was 2 cm beyond the lateral border of the popliteal artery, 20 cc of 0.2% ropivacaine combined with 5-mg preservative-free dexamethasone was injected after negative aspiration of blood (Figure 1). Due to the proximity of the common peroneal and tibial nerves, we decided to avoid using dexmedetomidine in the IPACK block. The safety of this combination has not been demonstrated in the IPACK or popliteal blocks.

**Figure 2:**
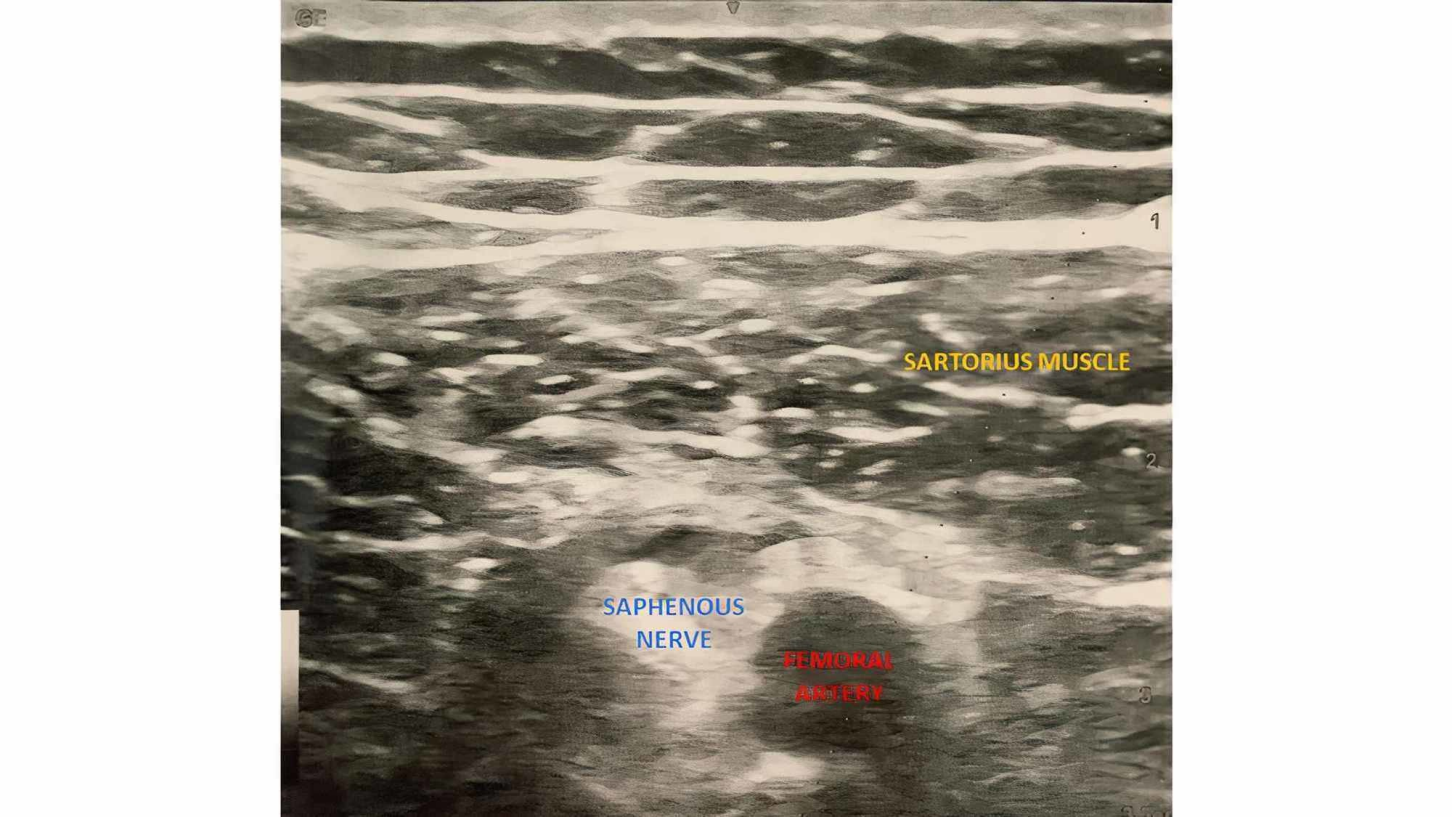
Gray-scale ultrasound with a linear probe showing the subsartorial space at the midthigh level. The sartorius muscle, saphenous nerve, and femoral artery can be seen.

With the patient in the supine position, the patient’s thigh was abducted and externally rotated to allow visualization and appropriate access to the medial thigh. The skin was disinfected, and the curvilinear probe was placed on the anteromedial thigh, at approximately the junction between the middle and distal third of the thigh. The femoral artery was identified and followed distally until it passed through the adductor hiatus, becoming the popliteal artery. The needle was inserted, in plane, in a lateral to medial orientation and advanced toward the femoral artery at the level of the mid-thigh. Once the needle tip was visualized anterior to the artery, 10 cc of the local anesthetic mixture of 25 mcg dexmedetomidine and 5-mg preservative-free dexamethasone was injected after negative aspiration of blood (Figure 2).

Case 1 

Consent to publish was obtained from all patients. The first case was a 54-year-old Caucasian male with a medical history of hypertension, 25 pack-year history of smoking, and obesity set to undergo a left TKA. The only opioid used during this patient’s general anesthetic was 100 mcg of fentanyl during the induction of anesthesia. The patient’s pain was a 3/10 in the post-anesthesia care unit following his procedure. Following discharge from the hospital, the patient reported analgesic efficacy lasting four days with progressively mild increase in pain on postoperative day 5. He subsequently did not require the use of opioid analgesics and used over-the-counter ibuprofen and acetamenophen to control his 5/10 pain once the effect of the blocks did subside.

Case 2 

Our second case was a 69-year-old African American female with a medical history of hypertension, sleep apnea with continuous positive airway pressure use, hypothyroidism, and morbid obesity undergoing a right TKA. The only opioid used during this patient’s general anesthetic was 100 mcg of fentanyl during the induction of anesthesia. The patient rated his pain as a 5/10 following her procedure, although she did not take any opioid analgesics. She reported five days of analgesia postoperatively with a mild increase of incisional pain on postoperative day 6.

Case 3

Our third patient was a 62-year-old African American male with a medical history of hypertension, 30 pack-year history of smoking, and mild chronic obstructive pulmonary disease who came in for a right TKA. The only opioid used during this patient’s general anesthetic was 100 mcg of fentanyl during the induction of anesthesia. On postoperative day 0, the patient rated his pain as a 4/10, although he stated he did not use any pain medication until the effect of the block subsided on postoperative day 4. He subsequently required 5 mg of oxycodone twice at night and once in the morning for two days until switching to acetaminophen for pain relief thereafter.

## Discussion

The posterior knee is not covered by femoral or ACBs. Other blocks such as the sciatic nerve block (SNB) may provide great relief for the posterior knee, but it has associated motor block of the lower extremity, limiting the ability to ambulate and move the knee through the range of motion. Furthermore, opioid requirements, length of stay, and distance walked on postoperative day 0 were all analyzed in a review of 100 TKA patients who received IPACK + ACB compared to femoral nerve block and SNB. It was concluded that the IPACK + ACB combination resulted in earlier ambulation, decreased opioid use, and decreased length of stay [6].

The IPACK + ACB may be a favorable combination of regional techniques for patients undergoing TKA. An ACB utilizing 0.75% ropivacaine has been demonstrated to provide approximately 10.8 hours of analgesia, with sparing of quadriceps motor function [7]. While many adjuvants to local anesthetics have been explored to prolong the duration of action of the utilized anesthetic, our series demonstrates the use of combined Dex-Dex in prolonging the duration of analgesia of the ACB blocks.

Dexmedetomidine is an alpha2-agonist that is utilized in regional anesthesia to prolong analgesia [8]. In a randomized controlled trial, dexmedetomidine added to 0.75% ropivacaine in an ACB was able to provide analgesia for 18.4 hours compared to a control group without dexmedetomidine only achieving 10.8 hours of analgesia. The mechanism of analgesic effect of alpha2-agonist such as dexmedetomidine stems from its action on hyperpolarization-activated cation currents in inhibiting the transmission of nociceptive fibers [9].

Dexamethasone has also been utilized in prolonging the action of local anesthetics. Its perineural mechanism, although likely multifactorial, may stem from its anti-inflammatory actions [10]. Dexamethasone was proven to prolong the duration of an ACB when compared to control (bupivacaine only) and decrease opioid consumption [11]. Few studies have utilized the combination of Dex-Dex. The combination, however, was previously proven to increase the analgesic duration of a caudal block prior to hypospadias surgeries in pediatrics [12]. Further studies could elucidate a possible synergistic effect of the two medications in prolonging the action of local anesthetics.

## Conclusions

The ACB + IPACK block offers excellent analgesia of the posterior knee with spared quadriceps muscle function allowing for early rehabilitation and discharge. The understanding of local anesthetic adjuvants and their potential is ever increasing. More studies are needed to understand a potential synergistic effect of Dex-Dex in safely prolonging the duration of analgesia obtained from regional anesthetic techniques and could someday explain how these patients achieved days of effective analgesia.
