# Correction: Adductor Canal Block Duration of Analgesia Successfully Prolonged With Perineural Dexmedetomidine and Dexamethasone in Addition to IPACK Block for Total Knee Arthroplasty

**DOI:** 10.7759/cureus.c39

**Published:** 2020-11-16

**Authors:** Jared Herman, Ivan Urits, Jonathan Eskander, Alan D Kaye, Omar Viswanath

**Affiliations:** 1 Anesthesiology, Mount Sinai Medical Center, Miami Beach , USA; 2 Anesthesiology, Critical Care, and Pain Medicine, Beth Israel Deaconess Medical Center, Harvard Medical School, Boston, USA; 3 Anesthesiology and Pain Medicine, Portsmouth Anesthesia Associates, Portsmouth, USA; 4 Department of Anesthesiology, Louisiana State University Shreveport, Shreveport, USA; 5 Pain Management, Valley Pain Consultants - Envision Physician Services, Phoenix, USA

This article was originally published with the fourth author, Alan D. Kaye, missing his middle initial. The author's name has been corrected to include the middle initial. Cureus regrets the error.

